# Supplementary material for: Determinants of preterm survival in a tertiary hospital in Ghana: A ten-year review
Source: PLoS One. 2021 Jan 22;16(1):e0246005. doi: 10.1371/journal.pone.0246005 (PMC7822519; doi:10.1371/journal.pone.0246005)
Supplement: Attachment — Submitted filename: Responses to Reviewer1-preterm study.docx [file pone.0246005.s002.docx]

Reviewer #1: Thank you for your revised manuscript entitled “Determinants of preterm survival in a tertiary hospital in Ghana: a ten-year review”
The revised manuscript shows much improvement by modifying the Tables, and I accept it after they revise two minor problems as below.

Minor problems
1. pp10, % of caesarean section;
In the revised manuscript, you showed the % of caesarean section among the 2254 babies as 45.5%. That is not correct. That should be 44.5%.

**Response**: We appreciate your keen sense of detail. This was a typographical error on our part.

Under Results/ 2^nd^ paragraph, the 1^st^ sentence of page 10 originally read as;

“Of those with the mode of delivery documented among the 2254 babies, 55.5% (1,100/1983) were vaginal and 45.5% (883/1983) were by caesarean section (see Table 1).”

It has now been corrected to read as below;

“Of those with the mode of delivery documented among the 2254 babies, 55.5% (1,100/1983) were vaginal and 44.5% (883/1983) were by caesarean section (see Table 1).”

2. Table1, Survival pf preterm neonates:
% of death should be 27.6%, not 29.0%. % of discharged/Alive should be 67.6%, not 71.0%.

**Response:** For this variable, data was available for 2144 preterm neonates AND NOT the total of 2254. The denominator is thus 2144 (this is shown beside the variable in the table). The percentages reported therefore stand correct (approximated to one decimal place).
