# Supplementary material for: Determinants of preterm survival in a tertiary hospital in Ghana: A ten-year review
Source: PLoS One. 2021 Jan 22;16(1):e0246005. doi: 10.1371/journal.pone.0246005 (PMC7822519; doi:10.1371/journal.pone.0246005)
Supplement: Attachment — Submitted filename: PRETERM STUDY RESPONSE TO EDITOR & REVIEWER COMMENTS.docx [file pone.0246005.s001.docx]

**RESPONSE TO EDITOR’S COMMENTS**

1. Please ensure that your manuscript meets PLOS ONE's style requirements, including those for file naming.

**Response**: In the current revision, we have adhered to the journal’s style and requirements to the best of our knowledge

2. In the ethics statement in the manuscript and in the online submission form, please provide additional information about the patient records used in your retrospective study, including: a) whether all data were fully anonymized before you accessed them; and b) the date range (month and year) during which patients' medical records were accessed. If patients provided informed written consent to have data from their medical records used in research, please include this information.

**Response:** the ‘data collection/handling’ section has been reviewed to reflect statements on [a] and [b] as well as the calibre of personnel used for the data collection and the training given. Similarly, the ‘ethical clearance’ section has been reviewed to include a statement on informed consent

- The original data collection/handling section read as;

Data on maternal age, parity, and mode and year of delivery, sex and weight of baby, gestational age, and 1^st^ and 5^th^ minute APGAR scores, length of stay and outcome (whether discharged alive or demised) were collected from the Admissions and Discharges book of the SCBU. This data was entered into an Excel spreadsheet, cleaned to exclude incomplete entries, then exported to SPSS version 22 for the analysis. Descriptive statistics (mean and frequency charts) were used, and chi-square and logistic regression employed to test for association between survival (defined as ‘being discharged home alive’) and its influencing factors.

It now reads as;

Data collection was from August 2018 to December 2019 and was conducted by two specialists- in-training (paediatrics and obstetrics/gynaecology). The principal investigator (EKA) trained the data collectors over three days on key components of the study including the objectives, inclusion/exclusion criteria and the study variables to be captured using a data extraction template.

Data on maternal and neonatal characteristics including maternal age, parity, and mode and year of delivery, sex and weight of baby, gestational age, and 1^st^ and 5^th^ minute APGAR scores, length of stay and outcome (whether discharged alive or demised) were collected from primary sources such as the admissions and discharges register of the SCBU, theatre and recovery unit registers and the birth register at the maternity ward. The data were anonymized by taking off personal identifiers and replacing them with study identity numbers. The data were entered into an Excel spreadsheet, cleaned to exclude incomplete entries and exported into SPSS version 22 for analysis. Data on 20% of the study participants were cross-checked with the primary sources for quality control.

Means and frequencies were computed and presented in tables and charts. Chi-square tests and logistic regression methods were employed to test for associations and the strength thereof between the dependent variable (preterm survival defined as ‘being discharged home alive’) and independent variables.

- The original ethical clearance section read as;

Ethical clearance with reference number **CCTHERC/EC/2018/20** was granted by the Ethical Review Board of CCTH.

It has been reviewed to now read as;

Ethical clearance for the study was granted by the ethical review board of CCTH with reference number CCTHERC/EC/2018/20. Since this was essentially a retrospective study using secondary data, it was not possible to obtain informed consent for the bulk of participants. Participants’ informed consent was therefore not sought. This was made clear in the application for ethical clearance.

3. We note you have included a table to which you do not refer in the text of your manuscript. Please ensure that you refer to Table 2 in your text; if accepted, production will need this reference to link the reader to the Table.

**Response:** Table 2 has been redone and presents the results of chi-square analysis for association between independent study variables and the survival outcomes

**RESPONSE TO REVIEWER #1 COMMENTS**

1. Introduction part is too long to be accepted. Authors should focus on what is the background and what the aim of their study is. They should describe them more clearly.

**Response:** The ‘introduction’ has been significantly revised and shortened. The study aim is also more clearly outlined.

2. Among 2,254 preterm babies, the authors pick up just 1,282 infants for the calculation of the prevalence of live preterm babies, because there was no precise information in 623 from referring facilities and 349 who don’t have any data of birth place. Then they might have excluded so many infants for calculation. I am not sure whether the prevalence of live preterm babies is meaningful data because there might be live preterm babies even among 623 and 349 excluded babies.

**Response**: The prevalence of live preterm births presented was for deliveries at the Cape Coast Teaching Hospital **ALONE**. The 623 were from other lower-level referring facilities and do not make any contribution to the prevalence measure.

It is possible there may have been some live preterm babies among the 349 for whom data on place of delivery was not documented as some of them may have been delivered at CCTH. It is again possible this could shoot up the prevalence as suggested but we cannot truly define what its impact would be. It would be acknowledged as a limitation and discussed.

Under ‘Results’, ‘Demographic and clinical characteristics of participants’ ……….the first paragraph originally read as;

“The study included a total of 2,254 preterm babies seen at the SCBU from 2010 to 2019. The CCTH had a total of 27,320 deliveries within that same period, out of which 1,282 were live preterm births, and 623 were from referring facilities whose total deliveries are not known, whiles data on place of birth of 349 babies were not documented. Thus, the prevalence of live preterm babies over the 10-year period at the CCTH alone was 4.7% (1,282/27,320). An increasing trend in prevalence is observed, with 2019 recording 9%, which is the highest for the study period (271/3027). [Figure 1.0].”

It is now revised to read as;

“The study included a total of 2,254 preterm babies seen at the SCBU from 2010 to 2019. The CCTH had a total of 27,320 deliveries within that same period, out of which 1,282 were live preterm births. A further 623 preterms were from different referring facilities while data on the place of birth for another 349 babies were not documented. Thus, the prevalence of live preterm babies over the 10-year period for deliveries at CCTH alone, was 4.7% (1,282/27,320)”

3. The number of live preterm babies were 1282, while that of survived were 1523.
   I am wondering why the number of survived is larger than that of live preterm babies.

**Response:** There is no confusion at all with both figures. The 1282 are the number of preterm babies **delivered** alive at the Cape Coast Teaching Hospital over the 10 year review period. The 1523, on the other hand, refer to the number of preterm babies **discharged** alive from the SCBU of the Cape Coast Teaching Hospital. The latter figure thus combines some of those delivered at CCTH and some of those referred from elsewhere to CCTH.

4. Table 1
1) The authors calculated % in each variable. but it is hard to understand at first looking, because it is not easy to find out how to calculate them. For example, In Male of “Sex of Baby”, they calculated 29.7% in Died, while 70.3% in Discharged alive, and finally sum of % is 100%. On the other hand, total number of Male should be 1115. But sum of 315 in Died and 746 in Discharged alive is not 1115, which makes me confused.

**Response:** We acknowledge the lack of clarity in Table 1. It has therefore be redone and presented more succinctly.

2) Page13: According to APGAR Score at 1 min in Table1, the prevalence of APGAR Score at 1min<4 is higher in “Discharged alive” than “Died” (136/355(38%) vs 205/355 (58%)). Is that true? The title of Table1 is “the Factors influencing the Survival of Preterm Babies”, and so your results mean that lower Apgar score at 1min is related to higher survival rate.

**Response:** We have presented results as obtained from our analysis**.**The results of the Chi square analysis for association between the categorical independent variable ‘Apgar Score at 1 min’ and categorical dependent variable ‘survival outcomes’ is now presented in Table 2. While the numbers pertaining to scores less than 4 are higher in the ‘discharged alive’ sub-category of the dependent variable, much higher numbers are found for scores higher than 4 and these underlie the significant association between ‘Apgar Score at 1 min’ and ‘survival outcomes’

5.Table3
1) In Table 3, the authors examined factors associated with the Survival of preterm babies and showed the Adjusted OR (95%CI). But I am afraid that their interpretation of results might be wrong.
2) According to Adjusted OR in Weight of Baby with Extremely low as reference (Page 16-17), increased birth weight is correlated with decreased survival rate. Moreover, according to Adjusted OR in APGAR Score at 5min with Score 1 as reference (Page 17-18), increased APGAR Score at 5 min is correlated with decreased survival rate. Those results should not make sense.
3) The results seem to be associated with death of preterm birth, not survival.

**Response:** Table 3 has been redone and the correlates speak to survival. For instance, increasing birth weight is correlated with higher odds of survival and so is the Apgar Score at 5 min.

**RESPONSE TO REVIEWER #2 COMMENTS**

1. Regarding the following two points:
   a) On page 5 “In Ghana, there is a strong association between preterm births and advanced maternal age (>35 years), suboptimal antenatal care, preeclampsia, antepartum haemorrhage, and preterm prelabour rupture of membranes (PPROM).”

   b) On page 10 “An increasing trend in prevalence is observed, with 2019 recording 9%, which is the highest for the study period (271/3027). [Figure 1.0].”

   The causes of the increased rate of preterm births in Ghana need to be considered. If advanced maternal age pregnancies are the cause, the older the mother is, the higher the child mortality rate should be. However, in this paper, there is no association between child mortality and maternal age.

**Response:** The statements referred to are from previous studies in Ghana and relate the incidence of preterm births to the listed factors including advanced maternal age. In the present study, we have sought to examine correlates between maternal age and survival of preterm infants ( a different phenomenon from incidence of preterm births). Our study did not find an association between maternal age and the preterm deaths/survival. There could be a number of reasons for this such as fewer numbers of women older than 35 years. It could also be that some confounders are at play here. The lack of an association will be discussed in the context of existing literature review.

2. There are small grammatical errors. Examples are listed below:
On page 2:
a) Special Care Baby unit: Special Care Baby Unit

b) Data on 2,254 babies that met the inclusion criteria was extracted: Data on 2,254 babies that met the inclusion criteria were extracted

c) Descriptive statistics were generated and test of association done with chi-square and multivariable logistic regression: Descriptive statistics were generated and tests of association done with chi-square and multivariable logistic regression

d) An increasing trend in prevalence is observed, with 2019 recording the highest of 9% (271/3027).: An increasing trend in prevalence was observed, with 2019 recording the highest at 9% (271/3027).

**Response:** These have been rectified as suggested.

3.On page 3:
e) It also highlighted the problem of inadequate data capture: It also highlights the problem of inadequate data capture

**Response:** Correction done as suggested

4.On page 7:
f) This study, therefore, will be the first to report on survival of preterm babies: This study, therefore, will be the first to report on the survival of preterm babies

**Response:** This has been revised to read as;

# “This study is the first to report on survival of preterm babies at a specialised neonatal care unit in Ghana.”

5.On page 8:
g) which lacks some of the logistics of neonatal intensive care unit (NICU): which lacks some of the logistics of a neonatal intensive care unit (NICU)

**Response:** correction done as suggested

6.On page 9:
h) Data on maternal age, parity, and mode and year of delivery, sex and weight of baby, gestational age, and 1st and 5th minute APGAR scores, length of stay and outcome (whether discharged alive or demised) were collected: Data on maternal age, parity, mode and year of delivery, sex and weight of the baby, gestational age, 1st and 5th minute APGAR scores, length of stay, and outcome (whether discharged alive or demised) were collected

**Response:** corrections done as suggested

7.On page 10:
i) whiles data on place of birth of 349 babies were not documented.: while data on the place of birth of 349 babies were not documented.

**Response:** corrected and reworded as;

“…………………………. while data on the place of birth for another 349 babies were not documented.”

8.There was no statistically significant relationship between survival and the sex of a neonate (p = 3.06), age of mother (p = 0.598), parity of mother (p = 0.717), year of delivery (p = 0.212) [Table1].: There was no statistically significant relationship between survival and the sex of a neonate (p = 3.06), age of mother (p = 0.598), parity of mother (p = 0.717), or year of delivery (p = 0.212) [Table1].

**Response:** The above has been revised to read as;

“survival of the preterm neonate was independent of the sex of baby, maternal age and parity (see Table 1).”

9.On page 11:
k) However, the weight of a baby (p = <0.001), referring point (p <0.001),: However, the weight of the baby (p = <0.001), referral point (p <0.001),

**Response:** correction done as suggested

10.On page 21:
l) as well as facilitate antenatal counselling on high-risk infants.: as well as facilitate antenatal counselling in high-risk infants.

**Response:** correction done as suggested

11.On page 22:
m) whiles an overall singleton preterm survival rate of 38.4% has been reported in Nigeria. [26]: while an overall singleton preterm survival rate of 38.4% has been reported in Nigeria. [26]

**Response:** corrected as suggested

12.On page 23:
o) Survival of preterm babies is comparatively very high in resource rich countries due to the readily-available technological equipment.: The survival of preterm babies is comparatively very high in resource rich countries due to readily-available technological equipment

**Response:** correction done as suggested

13.Mortality rates is 52%: Mortality rates are 52%

On page 24:
q) consumer-satisfaction: patient-satisfaction

r) We found an increasing prevalence of live preterm birth at CCTH,: We found an increasing prevalence of live preterm births at CCTH,

**Response**: all corrections done as suggested

14.s) Regarding the characteristics of the population, there was no significant difference between sex of baby and mortality.: Regarding the characteristics of the population, there was no significant difference between the sex of the baby and mortality.

**Response:** reworded to read “Regarding the characteristics of the population, there was no significant association between the sex of the baby and mortality
